# Computer-aided diagnosis with a convolutional neural network algorithm for automated detection of urinary tract stones on plain X-ray

**DOI:** 10.1186/s12894-021-00874-9

**Published:** 2021-08-05

**Authors:** Masaki Kobayashi, Junichiro Ishioka, Yoh Matsuoka, Yuichi Fukuda, Yusuke Kohno, Keizo Kawano, Shinji Morimoto, Rie Muta, Motohiro Fujiwara, Naoko Kawamura, Tetsuo Okuno, Soichiro Yoshida, Minato Yokoyama, Rumi Suda, Ryota Saiki, Kenji Suzuki, Itsuo Kumazawa, Yasuhisa Fujii

**Affiliations:** 1 Department of Urology, Tsuchiura Kyodo General Hospital, Tsuchiura, Japan; 2 Department of Urology, Tokyo Medical and Dental University, Tokyo, Japan; 3 Department of Urology, JA Toride Medical Center, Toride, Japan; 4 Department of Information and Communications Engineering, Tokyo Institute of Technology, Tokyo, Japan; 5 Laboratory for Future, Interdisciplinary Research of Science and Technology, Institute of Innovative Research, Tokyo Institute of Technology, Tokyo, Japan

**Keywords:** Artificial intelligence, Convolutional neural network, Deep learning, X-ray, Urinary tract stone, Urolithiasis

## Abstract

**Background:**

Recent increased use of medical images induces further burden of their interpretation for physicians. A plain X-ray is a low-cost examination that has low-dose radiation exposure and high availability, although diagnosing urolithiasis using this method is not always easy. Since the advent of a convolutional neural network via deep learning in the 2000s, computer-aided diagnosis (CAD) has had a great impact on automatic image analysis in the urological field. The objective of our study was to develop a CAD system with deep learning architecture to detect urinary tract stones on a plain X-ray and to evaluate the model’s accuracy.

**Methods:**

We collected plain X-ray images of 1017 patients with a radio-opaque upper urinary tract stone. X-ray images (n = 827 and 190) were used as the training and test data, respectively. We used a 17-layer Residual Network as a convolutional neural network architecture for patch-wise training. The training data were repeatedly used until the best model accuracy was achieved within 300 runs. The F score, which is a harmonic mean of the sensitivity and positive predictive value (PPV) and represents the balance of the accuracy, was measured to evaluate the model’s accuracy.

**Results:**

Using deep learning, we developed a CAD model that needed 110 ms to provide an answer for each X-ray image. The best F score was 0.752, and the sensitivity and PPV were 0.872 and 0.662, respectively. When limited to a proximal ureter stone, the sensitivity and PPV were 0.925 and 0.876, respectively, and they were the lowest at mid-ureter.

**Conclusion:**

CAD of a plain X-ray may be a promising method to detect radio-opaque urinary tract stones with satisfactory sensitivity although the PPV could still be improved. The CAD model detects urinary tract stones quickly and automatically and has the potential to become a helpful screening modality especially for primary care physicians for diagnosing urolithiasis. Further study using a higher volume of data would improve the diagnostic performance of CAD models to detect urinary tract stones on a plain X-ray.

## Background

Urolithiasis is a common disease. Non-contrast computed tomography (CT) has become the gold standard modality as an imaging examination for diagnosing urolithiasis because of its high accuracy, which is reportedly 92–100%, and its excellent ability to detect other acute flank pain conditions [1–3]. However, some problems have emerged. CT is generally an expensive examination compared with others such as intravenous urography [4], and it is not preferred for pregnant women or children [5, 6]. Therefore, the repetitive use of CT is unsuitable for follow-up based on cost-effectiveness and radiation exposure. To reduce radiation exposure, low-dose CT is a promising option [7]. However, CT is not necessarily available in small-sized medical institutions such as in a medical office [8] because a CT scanner is expensive and requires a high level of interpretation capability. Conversely, a plain X-ray is a low-cost examination that has low-dose radiation exposure and high availability, although its accuracy, which is reportedly 44–77%, is inferior to that of CT [9].

An increased use of medical images induces further burden of their interpretation for physicians. Recently, a considerable amount of evidence has demonstrated the utility of artificial intelligence for diagnostic imaging. A computer-aided diagnosis (CAD) has been especially receiving attention in recent years. A neural network is a machine learning model that was designed to mimic human neural systems. Since the advent of a convolutional neural network (CNN) via deep learning in the 2000s, which is an advanced form of a neural network, CAD accuracy has increased and CAD has had a great impact on automatic image analysis in the urological field, leading to some successful reports about the detection of prostate cancer on magnetic resonance imaging (MRI) or the differentiation of distal ureteral stones and pelvic phleboliths [10, 11]. A combination of X-ray and CAD has also become successful in improving the diagnostic ability for various diseases, although its efficacy for identifying urinary tract stones has not been studied [12, 13]. In this study, we developed a CAD algorithm with deep learning architecture to automatically detect urinary tract stones on a plain X-ray image and evaluated the efficacy of this new model.

## Methods

### Study design and datasets

**Fig. 1:**
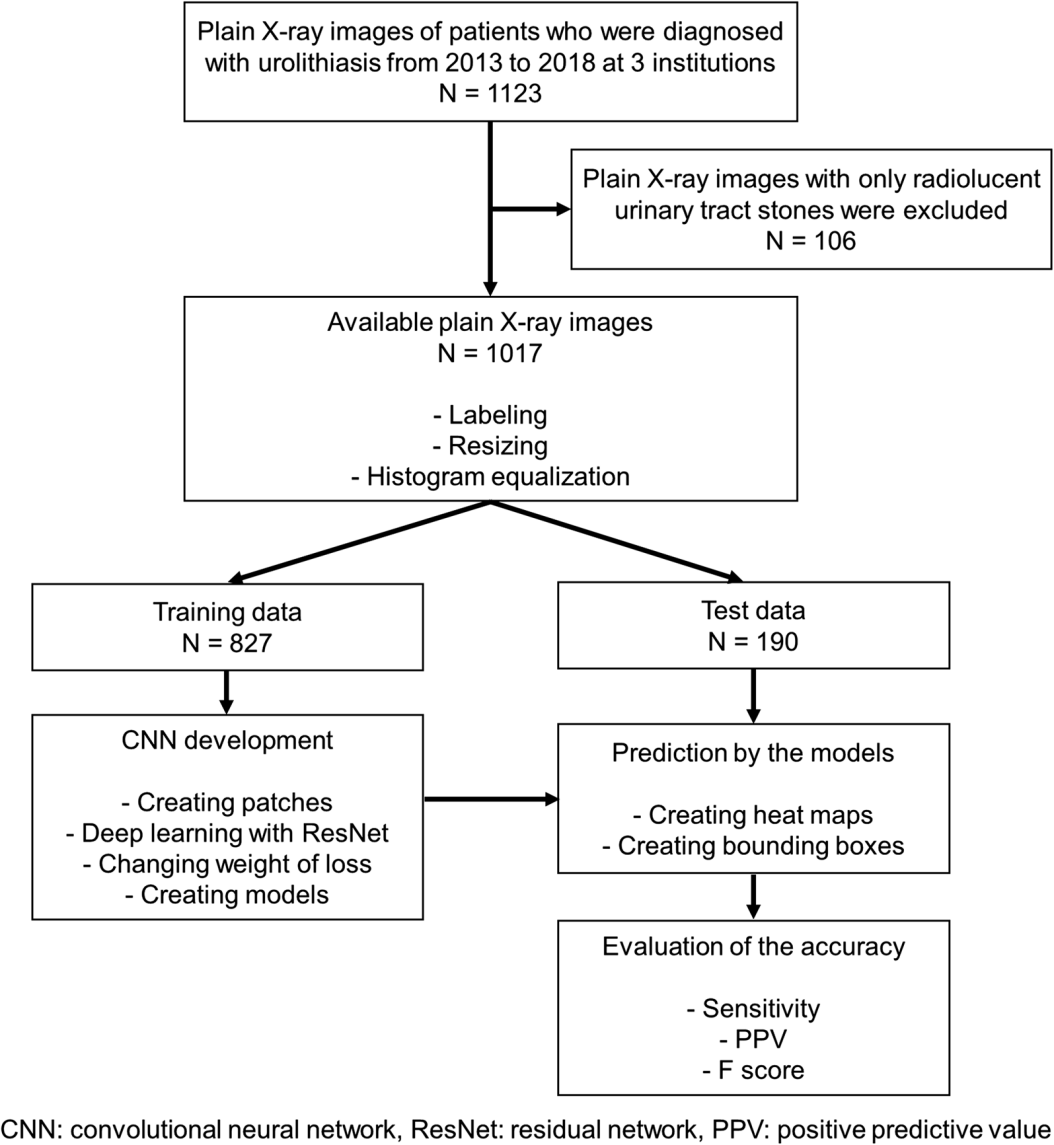
Flowchart of the inclusion and exclusion criteria and study outline. Eight hundred and twenty-seven X-ray images were used for training and 190 X-ray images were used for evaluating the model’s accuracy

Ethics board approval was obtained from the Tokyo Medical and Dental University Ethics Review Committee (approval number M2018-176). This was a multicenter retrospective study. We collected plain X-ray images of 1123 patients who were diagnosed with upper urinary tract urolithiasis from 2013 to 2018 at three institutions. The diagnosis of urolithiasis was confirmed by urologists based on the CT images in all cases. We excluded 106 cases that had only radiolucent urinary tract stones. X-ray images of 1017 patients with a radio-opaque urinary tract stone were used for this study. We enrolled 616 patients from Tsuchiura Kyodo General Hospital, 211 from Tokyo Medical and Dental University, and 190 from JA Toride Medical Center. We included only one image per patient. If the X-ray was repeated, only the image that was performed at diagnosis was included. Images with a visible artificial foreign body on the X-ray were included. We divided all X-ray images into two datasets, as follows: a training dataset consisting of 827 X-ray images from Tsuchiura Kyodo General Hospital and Tokyo Medical and Dental University, which were used to develop the CAD algorithm; and a test dataset consisting of 190 X-ray images from the JA Toride Medical Center, which were used to evaluate the model that we created. Figure 1 shows the inclusion and exclusion criteria and the outline of the present study.

### Labeling stone lesions

**Fig. 2:**
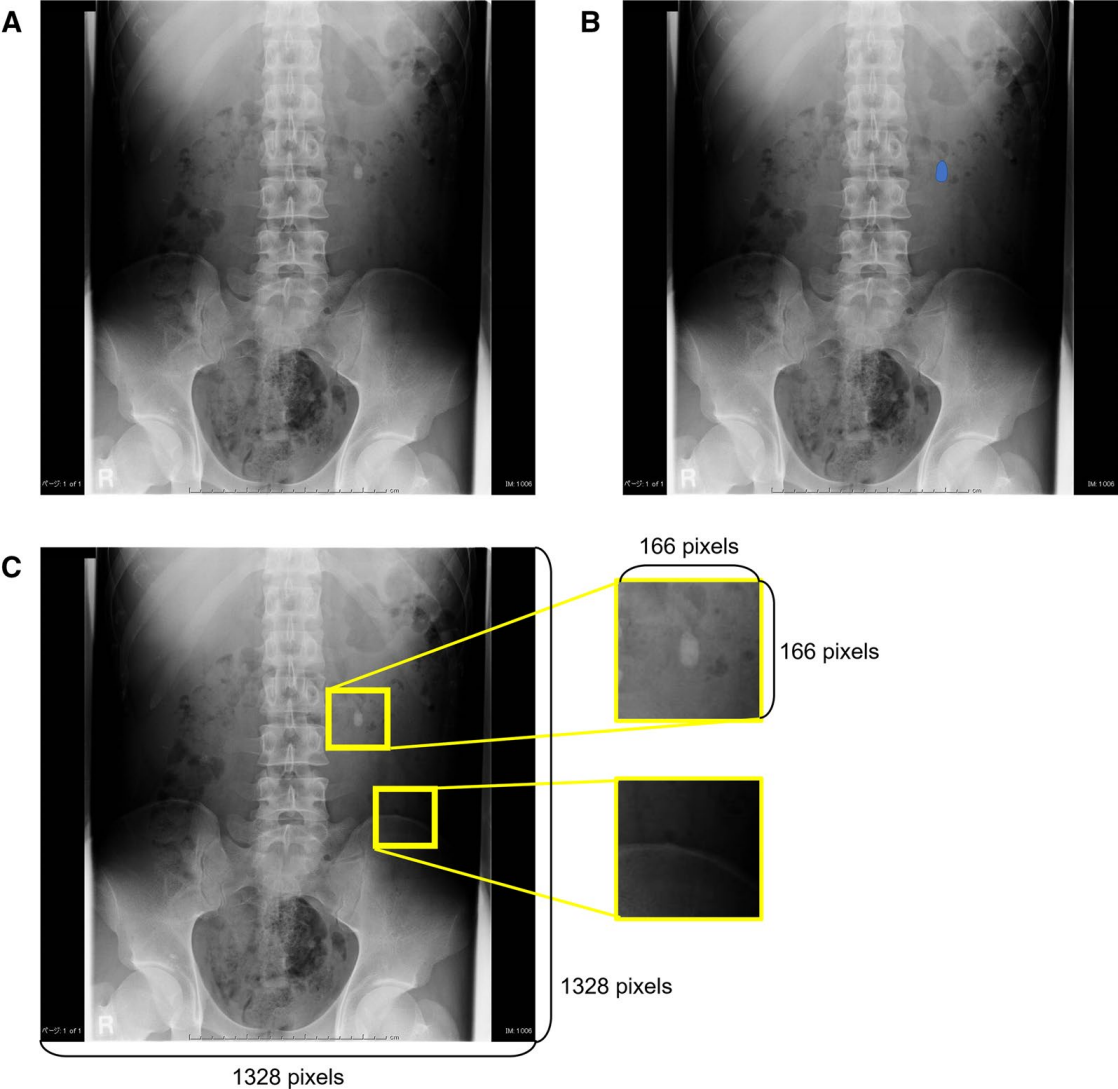
Labeling stone lesions and image division into patches. **a** Resized plain X-ray image of a patient with a left ureteral stone. **b** Labeling of stone lesions by urologists. A blue area in the image is a label showing the correct location of the stone lesion. **c** Random cropping and creating patches. Patches of 166 × 166 pixels were randomly cropped from a plain X-ray image and divided into two groups: patches including or not including a stone lesion

Among all of the X-ray images, all visible stone lesions were labeled as the correct information by urologists who manually traced the stone outline (Fig. 2a, b), with reference to the CT scan images. Invisible stone lesions on the X-ray were ignored in this study.

### Image pre-processing

All X-ray images were resized to 1328 × 1328 pixels to accommodate the different image size in each hospital. Histogram equalization was used to enhance contrast. In the training data, patches that were 166 × 166 pixels were cropped from resized X-ray images to randomly reduce the amount of calculations based on the following rules: (1) patches were forced to include a urinary tract stone or not at the same rate; (2) a urinary tract stone was forced to be centered in the patch if it was included; and (3) patches without any part of a urinary tract stone were forced to be created if the stone was not included (Fig. 2c). These patches were then divided into the following two groups based on whether or not the urinary tract stone lesion was contained in the patch: patches including or not including a stone lesion.

### Deep learning for training

**Fig. 3:**
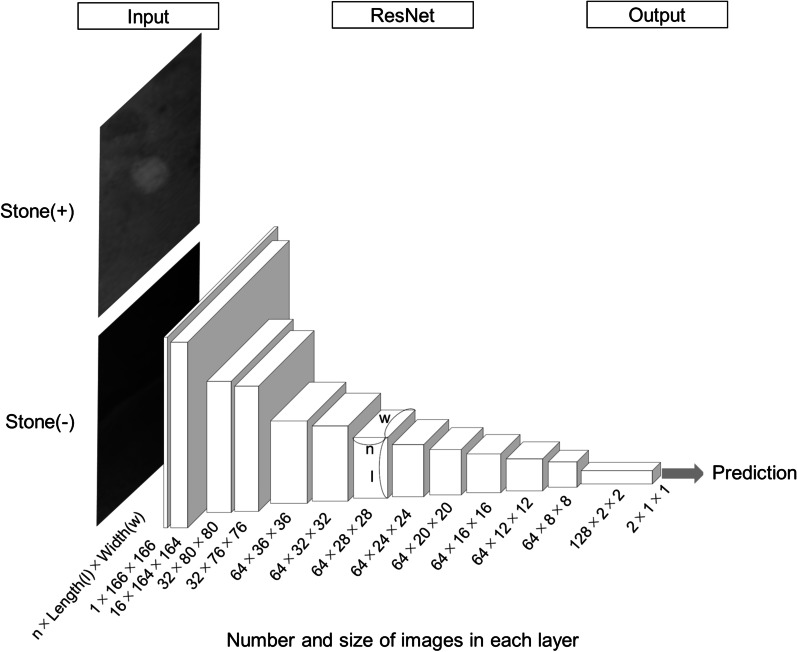
ResNet architecture. The patches were input and convoluted as they passed through each layer. Each box indicates the number (n) and size (length (l) × width (w) = pixels) of images in each layer. The computer’s prediction of whether an input patch was included was output and each loss was calculated if the output was not concordant with the input. The parameters were optimized using the back propagation method, in which each loss was supposed to be minimized

To develop the CAD algorithm, deep learning with graphics processing unit (GPU) computing using a computer equipped with GeForce GTX 1080 graphics card (Nvidia, Santa Clara, CA, USA) was performed. As a CNN architecture for a patch-wise training, we used a 17-layer Residual Network (ResNet) in this study [14]. Figure 3 shows the ResNet architecture. The patches including or not including a stone lesion were used as input data and the computer determined whether or not a stone lesion was contained in the patch. The outputs were normalized to show a probability of stone’s existence with the softmax function. There were two types of errors: overlooking and misdetection. An overlooking was an error that the patch included a stone lesion but the computer could not identify the stone, while a misdetection was an error that the patch included no stone lesion but the computer determined that a stone was present. Each error was calculated as a loss according to the probability of stone’s existence which the computer output as a result of prediction. Using the back propagation method, the CNN’s parameters were optimized to minimize each loss, which resulted in a model with fewer errors. In addition, several different weights of loss were tried for overlooking against misdetection to find a model with the most balanced diagnostic performance. The weight of loss for overlooking was a coefficient that we set up to adjust for the influence of overlooking when the weight of loss for misdetection was set to 1. For example, when the weight of loss for overlooking was set to 1, the loss was equally calculated among overlooking and misdetection. When the weight of loss for overlooking was set to 10, the loss for overlooking was calculated as 10 times more than that of misdetection. The weight of loss for overlooking affected how easily the model would overlook a stone lesion, and we could create models with different accuracies by changing this value. The training dataset was repeatedly used for the training, which was stopped when the model’s best accuracy was determined within 300 runs.

### Evaluation of the model’s accuracy

**Fig. 4:**
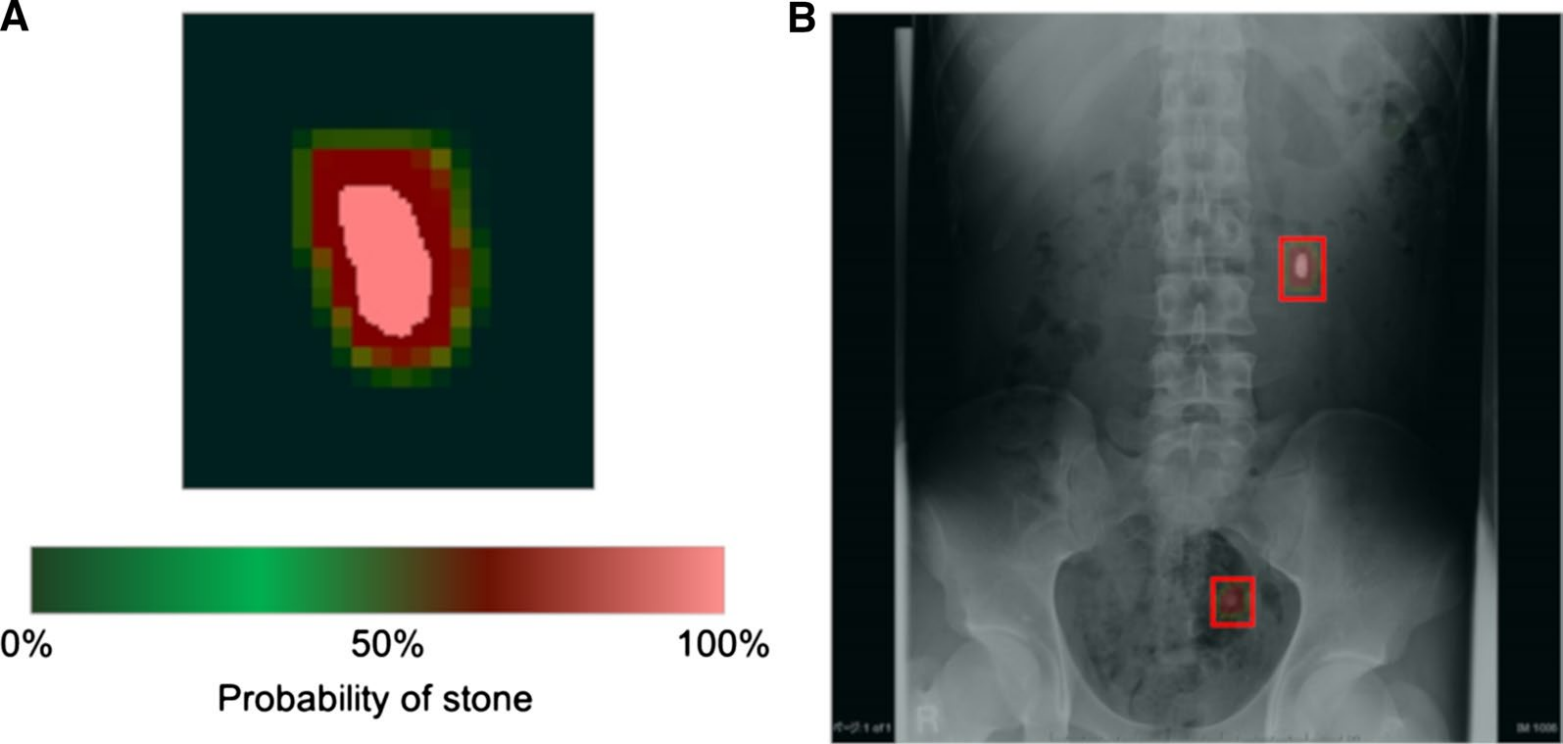
Preparation to evaluate the model’s accuracy. **a** Heat map representing the possibility of a stone lesion by color between light red at 100% and dark green at 0%. **b** Bounding boxes were automatically created to enclose three pixels outside of the heat maps

The test data were used to evaluate the accuracy of the model that we created using deep learning. The computer was forced to identify urinary tract stones in each whole X-ray image of the test data, not in the patches. As the result of prediction by the computer, the probability of stone’s existence was calculated in each pixel and demonstrated in each input image as heat maps that represented the probability of stone’s existence by the color between light red at 100% and dark green at 0% (Fig. 4a). In addition, bounding boxes were automatically created to enclose three pixels outside of the heat maps (Fig. 4b). If the bounding box completely contained the stone label, it was considered to be a true positive (TP), and if not, it was considered to be a false positive (FP). Additionally, if a stone lesion was ignored, it was considered to be a false negative (FN). TP or FP were assigned to each bounding box and FN to each ignored stone lesion, and they were then counted. We measured the sensitivity, positive predictive value (PPV), and F score of the models that we created, with three different weights of loss for overlooking, as follows: 1, 10, and 20. The F score, which is usually used to compare the models’ accuracies, is the harmonic mean of the sensitivity and PPV, and it reaches its maximum value at one and its worst value at zero. The closer the F score is to one, the more balanced is the diagnostic performance. Sensitivity, PPV, and the F score were calculated using the formulas that are described below.

$$\begin{aligned} & Sensitivity = \frac{TP}{{TP + FN}} \\ & Positive\;predictive\;value\;\left( {PPV} \right) = \frac{TP}{{TP + FP}} \\ & F\;score = \frac{2 \times Sensitivity \times PPV}{{Sensitivity + PPV}} \\ \end{aligned}$$

### Statistical analyses

Statistical analyses were performed using the JMP software program, version 10.0 (SAS, Cary, NC, USA). Data were compared between each group using Pearson’s chi-square test or Fisher’s exact test for categorical variables and Student’s t-test or Mann–Whitney U test for continuous variables. Chi-square test was also used for comparing sensitivity or PPV. *P* < 0.05 was considered statistically significant.

## Results

### Patients and images characteristics

The characteristics of patients and images that are assigned to the training and test datasets are summarized in Table 1. There were no significant differences between both datasets for gender and the number of labeled lesions per image (*P* = 0.132 and 0.486, respectively). Patients in the training dataset were significantly older than those in the test dataset (58 vs. 56 years old, *P* = 0.038). The proportion of images with a urinary tract stone located at mid-ureter was significantly lower in the training dataset than in the test dataset (9.1% vs. 14.2%, *P* = 0.046). However, the proportion of images with a urinary tract stone that was located in the distal ureter was significantly higher in the training dataset than in the test dataset (22.2% vs. 9.5%, *P* < 0.001). In the present study, images with an artificial foreign body such as a ureteral stent, a nephrostomy tube, an artificial joint, and a screw for spinal surgery were included, resulting in no significant differences between the training and test datasets (6.4% vs. 5.3%, *P* = 0.672).

**Table 1:** Characteristics of patients and X-ray images assigned to the training and test datasets

	Training dataset	Test dataset	*P* value
Number of patients	827	190	
Gender, n (%)			0.132
Male	537 (64.9)	143 (75.3)	
Female	290 (35.1)	47 (24.7)	
Age, median (range), years	58 (17–89)	56 (14–87)	0.038
Number of labeled lesions per image, n (%)			0.486
One	656 (79.4)	144 (75.8)	
Two	112 (13.5)	32 (16.8)	
More than two	59 (7.1)	14 (7.4)	
Location of urinary tract stone, n (%)			
Kidney	428 (51.8)	106 (55.8)	0.895
Proximal ureter	334 (40.4)	72 (37.9)	0.582
Mid-ureter	75 (9.1)	27 (14.2)	0.046
Distal ureter	184 (22.2)	18 (9.5)	< 0.001
Staghorn calculus, n (%)			0.553
Yes	17 (2.1)	2 (1.1)	
No	810 (97.9)	188 (98.9)	
Artificial foreign body in image, n (%)			0.672
Yes	53 (6.4)	10 (5.3)	
No	774 (93.6)	180 (94.7)	

### Deep learning results and the model’s accuracy

**Fig. 5:**
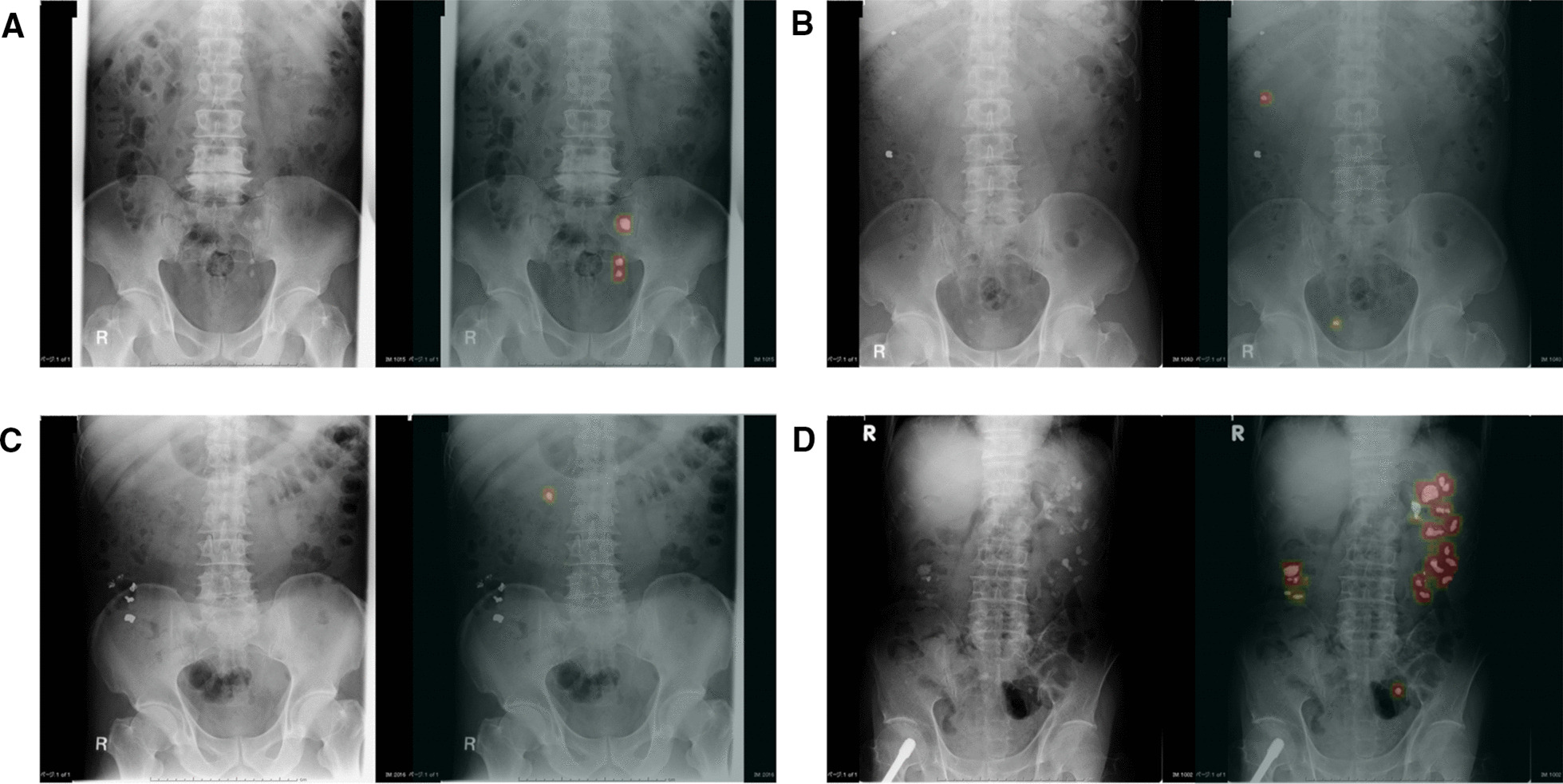
Visualization of four representative cases. **a** A case with multiple calculi including a mid-ureteral stone. **b** A case in which a calculus was able to be distinguished from pelvic phleboliths. **c** A case with residual barium in the colon. **d** A case with multiple calculi and an artificial joint

**Fig. 6:**
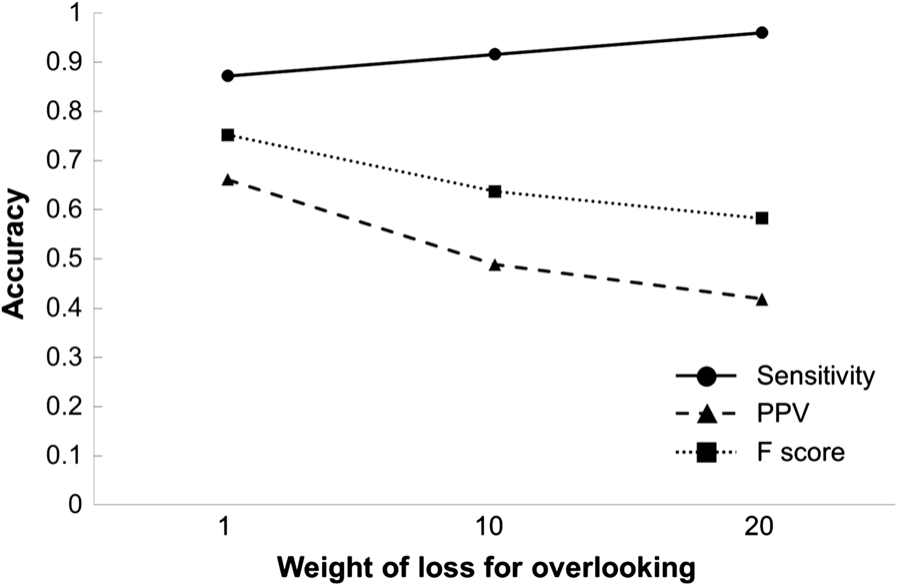
The models’ diagnostic performance that was created for each weight of loss for overlooking. This line graph indicates that the sensitivity was increased and that the PPV and F score were decreased as the weight of loss for overlooking was increased

The GPU needed 9 h to finish learning the training data. The model that we created needed 110 ms to provide an answer for each image. Figure 5 shows four representative cases in which the computer’s predictions were visualized. The sensitivity when the weights of loss for overlooking were 1, 10, and 20 was 0.872, 0.916, and 0.960, respectively. For each weight of loss, the PPV was 0.662, 0.488, and 0.418, and the F score was 0.752, 0.637, and 0.582, respectively. Figure 6 demonstrates a line graph showing the models’ diagnostic performances that was created for each weight of loss for overlooking, indicating that the sensitivity was increased and the PPV and F score were decreased as the weight of loss for overlooking increased. Based on the F score, the diagnostic performance of the model that was created when weight of loss for overlooking was set to 1 was considered to be the most balanced.

### The model accuracy for each urinary tract stone location

In our test dataset of 190 patients, 106 kidney stones, 107 proximal ureteral stones, 22 mid-ureteral stones, and 30 distal ureteral stones were identified by urologists. We studied the differences in the model’s diagnostic performance based on the location of a urinary tract stone, using the model that was created when the weight of loss for overlooking was set to 1. In this case, the CAD model was able to detect 95 stones in the kidney, 99 stones in the proximal ureter, 13 at mid-ureter, and 24 in the distal ureter. Conversely, there were 72 FPs in the kidney, 14 in the proximal ureter, 13 in the mid-ureter, and 19 in the distal ureter. The diagnostic performance was as follows: the sensitivity was 0.896 in the kidney, 0.925 in the proximal ureter, 0.591 at mid-ureter, and 0.800 in the distal ureter. The sensitivity for mid-ureter stone was significantly lower than that for kidney and proximal ureter stone (*P* = 0.001 and < 0.001, respectively). The PPV was 0.569 in the kidney, 0.876 in the proximal ureter, 0.500 at mid-ureter, and 0.558 in the distal ureter (Table 2). The PPV for proximal ureter was significantly higher than that for kidney, mid-ureter and distal ureter stone (*P* < 0.001, < 0.001, and < 0.001, respectively). There were no significant differences except for the above-mentioned comparisons.

**Table 2:** The accuracy of the model for each urinary tract stone location

Urinary tract stone location	Number of TP	Number of FP	Number of FN	Sensitivity	PPV
Kidney	95	72	11	0.896	0.569
Proximal ureter	99	14	8	0.925	0.876
Mid-ureter	13	13	9	0.591	0.500
Distal ureter	24	19	6	0.800	0.558
All locations	231	118	34	0.872	0.662

*TP* true positive, *FP* false positive, *FN* false negative, *PPV* positive predictive value

## Discussion

In this study, we used deep learning to develop a CAD system to detect radio-opaque urinary tract stones on a plain X-ray. To our knowledge, there has been no report about automatic detection of urinary tract stones on plain X-ray. Our model was able to detect urinary tract stones quickly with a high sensitivity. This might become a new screening modality for diagnosing urolithiasis.

Automatic detection of urinary tract stones using a CAD system has several advantages. First, a CAD system is always able to provide quick and consistent interpretation without fatigue or fickleness. This capability is helpful in emergency medicine. Second, a CAD system is economical and easy to access because it can function as an application on a computer. There is no need to purchase any special machine and all we need to do is to install the application. Third, a CAD system with deep learning can have a self-learning system. Learning the mistakes that were made using a CAD system allows improvement of its diagnostic performance. Given these advantages, a plain X-ray with a CAD system may become a reliable modality in clinical practice as a screening tool, although the main problem is its accuracy.

We studied the diagnostic performance of a CAD system with a focus on a balance by changing the weight of loss for overlooking. In this study, we measured the F score, which represented a balance of the CAD system’s diagnostic performance between overlooking and misdetection. The F score was best when the weight of loss for overlooking was set to 1, and the sensitivity and PPV were 0.872 and 0.662. According to the previous report about automatic detection of abnormality on chest X-ray, the sensitivity and specificity were 0.887 and 0.696, which are similar to our results [13]. The sensitivity seemed to be so satisfactory that the CAD system could help primary care physicians to find a urinary tract stone. In fact, this CAD model was able to detect a kidney stone of 2 mm in diameter (Fig. 5b). However, PPV was low, particularly in the kidney, at mid-ureter, and in the distal ureter. It was probably because the computer was likely to misdiagnose calcifications or bones as urinary tract stones. The computer did not have knowledge about the structure of the human body, resulting in mistakes that would not be made by physicians. On the other hand, another study reported that the combination of CAD system and CT had high accuracy in automatic differentiation of distal ureteral stones and pelvic phleboliths with the sensitivity of 0.94 and the specificity of 0.9 [11]. Another possible reason is the small amount of training data that we used in this study. We prepared only 1017 images for deep learning. This amount was low for training data for adequate deep learning. For example, in a previous report about deep learning for chest X-ray, over 100,000 images were used [15]. In the future, if we are able to prepare an adequate amount of training data and combine it with another algorithm to identify the human body’s structure, the accuracy may be further enhanced. The CAD system that we created in this study seems to be useful as a screening tool, because X-ray has the several advantages such as low-cost, low-dose radiation and high availability.

Data augmentation is a method that is used to amplify training data by adding a change to an original image. We did not perform data augmentation in this study. In our pilot analysis, data augmentation did not improve accuracy (i.e. the best F score was 0.636), which was probably because the computer identified urinary tract stones based on their shape or orientation. Data augmentation by transformation or rotation might not be helpful for developing the CAD system to detect urinary tract stones. However, data augmentation without transformation or rotation may improve the accuracy. One such method is embedding augmentation, which means making a fake image that is similar to a real image [16]. Using stone embedding, we can create a large amount of fake images of urolithiasis and increase the training data for deep learning, leading to an improvement in the CAD system’s diagnostic performance.

The present study has several limitations. First, we excluded cases with only radiolucent urinary tract stones, which is reportedly observed in 10% of patients with urolithiasis, because the diagnostic ability of the algorithm depends on the contrast information in the image [17]. This model can identify only lesions visible on plain X-ray. A radiolucent urinary tract stone will not be detected by models using a plain X-ray trained with CNN algorithm, although the combination with ultrasonography or intravenous pyelogram may be effective. Although the diagnostic performance of a CAD system would currently be inferior to CT for diagnosing radiolucent stones, a CAD system could be improved in the future, and it is expected to be used in various situations such as follow-up until stone expulsion or objective evaluation of stone treatment. In addition, if trained based on CT or MRI, the CAD system seems to gain the ability to accommodate various types of lesions, as we reported about automatic detection of prostate cancer in the previous study [10]. The efficacy of a CAD system combined with CT or MRI images for the urological field including not only urolithiasis but also neoplastic disease is under investigation. Second, the proportion of X-ray images with a distal ureteral stone was lower in the test dataset than in the training dataset. Distal ureteral stones were difficult to distinguish from pelvic phleboliths, which might tend to increase the number of FP results. Therefore, the test dataset in the present study might contribute to reducing the number of FPs. Third, we did not include negative images without a stone lesion. Therefore, the specificity and negative predictive value could not be calculated in this study. Prospective investigations of the CAD system’s usefulness are required for urinary stone clinical practice.

## Conclusions

We developed a CAD system using deep learning to detect radio-opaque urinary tract stones on a plain X-ray. The sensitivity was high enough to use the CAD model in clinical practice, although there was still room to improve the PPV. The CAD model can detect urinary tract stones quickly and automatically, and has the potential to help primary care physicians to diagnose urolithiasis as a screening modality. Further study using higher-volume data would improve the CAD models’ diagnostic performance for detecting urinary tract stones on a plain X-ray.

## Data Availability

The data that support the findings of this study are available on request from the corresponding author. The data are not publicly available because the data contain information that could compromise research participant privacy.

## Abbreviations

CAD: Computer-aided diagnosis
CNN: Convolutional neural network
CT: Computed tomography
FN: False negative
FP: False positive
GPU: Graphics processing unit
MRI: Magnetic resonance imaging
PPV: Positive predictive value
ResNet: Residual network
TP: True positive
